# Incorporating Next-Generation Sequencing in Newborn Screening for Organic Acidemias

**DOI:** 10.3390/ijns12010018

**Published:** 2026-03-19

**Authors:** Yiming Lin, Jinping Zhong, Weilin Peng, Faming Zheng, Xudong Wang

**Affiliations:** 1 Department of Clinical Laboratory, Maternity and Children’s Hospital, School of Medicine, Huaqiao University, Quanzhou 362021, China; zjp197901@163.com (J.Z.); wellpeng@163.com (W.P.); 22192325@163.com (F.Z.); 2 Center of Neonatal Disease Screening, Maternity and Children’s Hospital, School of Medicine, Huaqiao University, Quanzhou 362021, China; 3 Xiamen Newborn Screening Center, Department of Pediatrics, Women and Children’s Hospital, School of Medicine, Xiamen University, Xiamen 361102, China

**Keywords:** organic acidemias, newborn screening, next generation sequencing, combined genetic screening

## Abstract

Organic acidemias (OADs) are a group of inherited metabolic disorders with a high false-positive rate in newborn screening. In this study, we aimed to evaluate the clinical performance of next-generation sequencing (NGS) as a combined genetic test for OADs. From September 2022 to August 2025, 154,634 newborns underwent primary screening using tandem mass spectrometry (MS/MS). Among them, 151 neonates with suspected OADs underwent combined genetic screening using a pre-designed NGS panel. Of these, 55 cases tested positive on genetic screening, and 17 were ultimately diagnosed with OADs, yielding a prevalence of 1 in 9096. The positive predictive value of NGS was 30.91% (17/55). The genotypes of nine patients (9/17, 52.9%) were identified through NGS screening. Notably, one case of methylmalonic acidemia that would have been missed by MS/MS screening was successfully identified using the combined genetic screening. Additionally, 37 neonates with positive biochemical screening results were confirmed to be either carriers or unaffected individuals. Two cases of Wilson’s disease were also identified through combined genetic screening. Therefore, integrating NGS into conventional MS/MS-based screening can significantly reduce the false-positive rate and shorten the time from screening to definitive diagnosis. This approach provides a valuable model for improving the efficiency and accuracy of newborn genetic screening.

## 1. Introduction

Newborn screening (NBS) has become an effective public health program worldwide over the past few decades, enabling the identification of various serious but treatable inherited and metabolic diseases in newborns [1]. It facilitates early diagnosis and timely intervention, significantly improving the long-term outcomes of affected neonates [2]. Tandem mass spectrometry (MS/MS) has been widely integrated into NBS programs. This technology greatly expands the scope of disease detection by enabling the simultaneous quantification of amino acids, acylcarnitines and succinylacetone in neonatal dried blood spot (DBS) samples. Numerous inherited metabolic disorders (IMDs), including amino acid disorders, organic acidemias (OADs), and fatty acid oxidation defects, can be detected using MS/MS [3,4,5]. MS/MS-based biochemical screening primarily relies on the measurement of metabolic biomarkers; therefore, only diseases with specific biomarkers can be included in NBS panels. Moreover, traditional biochemical screening has been criticized for its high false-positive rate (FPR), low positive predictive value (PPV), unnecessary recalls for retesting, and the need for confirmatory diagnostic testing [6,7,8].

To improve the efficiency of NBS, genetic screening has been proposed as an adjunct to traditional NBS. This approach can reduce unnecessary recalls for secondary DBS collection and effectively shorten the turnaround time from screening to diagnosis and treatment. Fluorescent quantitative PCR and MassARRAY nucleic acid mass spectrometry are commonly used to screen for common pathogenic variants in specific diseases, such as primary carnitine deficiency, multiple acyl-CoA dehydrogenase deficiency, and citrin deficiency [9,10,11,12]. With technological advances and declining sequencing costs, an increasing number of studies have applied next-generation sequencing (NGS) in NBS programs [13,14,15,16,17]. Although NGS-based newborn screening offers advantages in high throughput and broad disease coverage, its optimal implementation remains under debate [18,19]. Some researchers advocate universal genomic screening for all newborns, whereas others argue that sequencing should be reserved for those with positive primary NBS results, as it may be unnecessary for screen-negative newborns [20,21,22,23]. Organic acidemias (OADs) are a group of IMDs detectable by MS/MS but are associated with relatively high FPR. Therefore, genetic testing of newborns with abnormal NBS results may be warranted. To evaluate the clinical feasibility of NGS, we conducted a pilot study implementing combined genetic screening in newborns with NBS results highly suggestive of OADs.

## 2. Materials and Methods

### 2.1. Study Design and Participants

**Figure 1:**
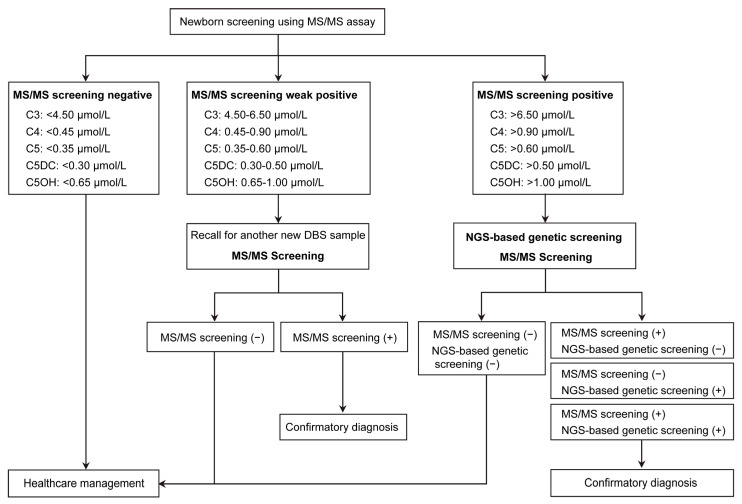
Newborn screening algorithm of combined genetic screening. C3: propionylcarnitine; C4: butyrylcarnitine; C5: isovalerylcarnitine; C5DC: glutarylcarnitine; C5OH: 3-hydroxyisovalerylcarnitine; MS/MS, tandem mass spectrometry (MS/MS); +, positive; −, negative.

Since 2014, the Newborn Screening Laboratory of Quanzhou Maternity and Children’s Hospital has provided NBS services for 27 IMDs to all newborns born in Quanzhou, Fujian Province, China [24]. Between 1 September 2022, and 31 August 2025, first-tier biochemical screening using MS/MS was performed in 154,634 newborns. In the traditional NBS algorithm, further diagnostic analysis is performed for all newborns with positive retest results. In this study, a higher cutoff value was established to determine eligibility for combined genetic screening, thereby excluding newborns at low risk for OADs. Newborns with suspected OADs were referred for combined genetic screening (Figure 1). Because other OADs are extremely rare in our population, we selected five acylcarnitines as biomarkers for genetic screening. Newborns with any of the following biomarkers, propionylcarnitine (C3), butyrylcarnitine (C4), isovalerylcarnitine (C5), glutarylcarnitine (C5DC), or 3-hydroxyisovalerylcarnitine (C5OH), exceeding the predefined genetic screening cutoff were eligible for combined genetic testing. The cutoff values for genetic screening were determined primarily based on concentrations observed in conformed local cases and data reported in the literature. The cutoff value was set at approximately twice the upper limit of the traditional cutoff value and further adjusted for each specific disease (Table 1). Preterm newborns (gestational age < 37 weeks) and/or low-birth-weight newborns (<2500 g) were excluded.

This study was approved by the Ethics Committee of Quanzhou Maternity and Children’s Hospital (reference number: 2024-IRB-107). Written informed consent was obtained from the parents or legal guardians. All relevant information regarding the screened diseases, limitations of screening methods, and potential risks of combined genetic screening was fully disclosed to the guardians. Genetic screening reports were provided to families when pathogenic or likely pathogenic variants closely associated with the five aforementioned biomarkers were identified. Additionally, genetic screening reports were provided when two variants were detected in genes associated with recessive genetic diseases or when one variant was detected in a gene associated with a dominant genetic disease. Further diagnostic analysis was recommended for all affected families.

### 2.2. First-Tier Biochemical Screening for OADs

Neonatal heel blood specimens were collected from all neonates between 3 and 7 days after birth. The samples were dried at room temperature (18–25 °C) and transported to the Newborn Screening Laboratory via cold-chain logistics within 3 days. According to the manufacturer’s instructions, samples were pre-processed using the NeoBase™ non-derivatized MS/MS reagent kit (PerkinElmer, Turku, Finland) and analyzed using AQCUTY TQD and TQS Micro tandem mass spectrometers (Waters, Milford, MA, USA). Newborns with abnormal MS/MS results exceeding the NBS cutoff but below the predefined genetic screening cutoff were recalled for repeat DBS collection and retesting. Newborns with MS/MS results exceeding the genetic screening cutoff value for genetic screening were referred for combined genetic screening. All newborns with positive results on repeat biochemical or genetic screening underwent further confirmatory diagnostic analysis (Figure 1).

### 2.3. Combined Genetic Screening for OADs

In this study, a pre-designed NGS panel [25,26] was used for combined genetic screening. The panel targeted more than 2800 pathogenic variants across 135 disease-causing genes associated with 75 neonatal genetic disorders (Supplemental File: Table S1). Genomic DNA was extracted from residual DBS samples using an automated nucleic acid extraction system (Bioer, Hangzhou, China). DNA libraries were constructed using the stem-loop inhibition-mediated amplification (SLIMamp) method, a multiplex PCR-based technique. Library quality was evaluated using an Agilent Bioanalyzer 2100 (Agilent Technologies, Santa Clara, CA, USA). High-throughput sequencing was performed on the Illumina NextSeq 500 platform (Illumina, San Diego, CA, USA) according to the manufacturer’s instructions.

### 2.4. Confirmatory Genetic Analysis and Diagnosis of OADs

Screen negativity was defined as negative results on both MS/MS screening and genetic screening. A neonate was considered screen-positive if either the MS/MS screening and/or genetic screening result was positive. All screen-positive neonates were transferred to specialists for confirmatory genetic testing and clinical evaluation. Confirmatory genetic analysis was performed using targeted capture-based NGS, as previously described [27], covering the exons and flanking regions of 107 genes associated with IMDs (Supplemental File: Table S2). All candidate disease-causing variants were confirmed using Sanger sequencing. Newborns harboring biallelic pathogenic/likely pathogenic variants in genes associated with OADs were diagnosed as OAD-positive. Newborns with one or no pathogenic/likely pathogenic variants identified on combined genetic screening were excluded without further intervention.

## 3. Results

### 3.1. Overview of Combined Screening Results

**Figure 2:**
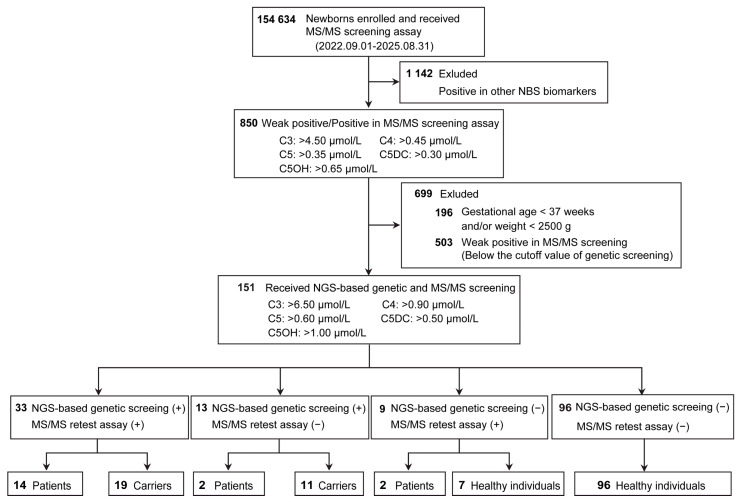
Summary of combined screening results. C3: propionylcarnitine; C4: butyrylcarnitine; C5: isovalerylcarnitine; C5DC: glutarylcarnitine; C5OH: 3-hydroxyisovalerylcarnitine; MS/MS, tandem mass spectrometry (MS/MS); NGS, next-generation sequencing; +, positive; −, negative.

Among 154,634 newborns, 1992 screened positive on first-tier biochemical screening. Of these, 850 were suspected of having OADs. NGS-based genetic screening was performed in 151 newborns, including 45 with elevated C3 levels, 21 with elevated C4, 61 with elevated C5, 4 with elevated C5DC, and 20 with elevated C5OH. Fifty-five newborns tested positive on combined genetic screening, and 17 were finally diagnosed with OADs, resulting in a prevalence of 1 in 9096 (Figure 2). In the present study, the genotypes of nine patients (52.9%; P1, P3, P5, P10-P14, and P16) were identified through combined genetic screening. In addition, two neonates were diagnosed with Wilson’s disease (WD; OMIM# 277900) through combined genetic screening (Table 2). Ultimately, the PPV for OADs was 30.9% (17/55) with the implementation of combined genetic screening.

### 3.2. Confirmatory Diagnosis of Screen-Positive Patients

Seven types of OADs were identified in 17 patients, including three with methylmalonic acidemia (MMA, OMIM #609058), three with 3-methylcrotonyl-CoA carboxylase deficiency (3-MCCD, OMIM #210210), three with isobutyryl-CoA dehydrogenase deficiency (IBDHD, OMIM #611283), three with 2-Methylbutyryl-CoA dehydrogenase deficiency (2-MBAD, OMIM #600301), two with short-chain acyl-CoA dehydrogenase deficiency (SCADD, OMIM #201470), two with glutaric acidemia type I (GA-I, OMIM #231670), and one with isovaleric acidemia (IVA, OMIM #243500). Biochemical retesting showed that all patients except one (P3) remained positive on repeat DBS testing. Two patients (P17 and P18) were positive on repeat biochemical testing but negative on combined genetic testing. Furthermore, two patients (P4 and P7) were diagnosed with Wilson’s disease via combined genetic screening.

**Table 2:** Diagnosis of positive patients using tandem mass spectrometry combined with genetic screening.

No.	Gender	NBS (μmol/L)	Retest (μmol/L) *	Combined Genetic Screening	Confirmatory Genetic Test (Targeted NGS and Sanger Sequencing)	Disorders
P1	Female	C3: 6.67	C3: 12.8	*MMUT*: c.[1663G>A]; [1106G>A] or p.[Ala555Thr]; [Arg369His]	*MMUT*: c.[1663G>A]; [1106G>A] or p.[Ala555Thr]; [Arg369His]	MMA
P2	Male	C3: 9.04	C3: 14.93	*MMUT*: c.[1777G>T] or p.[Glu593*]	*MMUT*: c.[1777G>T]; [1159A>C] or p.[Glu593*]; [Thr387Pro]	MMA
P3	Female	C3: 6.78	**C3: 2.43**	*MMUT*: c.[914T>C]; [544dup] or p.[Leu305Ser]; [Met182Asnfs*29]	*MMUT*: c.[914T>C]; [544dup] or p.[Leu305Ser]; [Met182Asnfs*29]	MMA
P4	Female	C3: 8.72	**C3: 0.55**	*ATP7B*: c.[2333G>T]; [2333G>T] or p.[Arg778Leu]; [Arg778Leu]	*ATP7B*: c.[2333G>T]; [2333G>T] or p.[Arg778Leu]; [Arg778Leu]	WD
P5	Female	C4: 1.48	C4: 1.81	*ACADS*: c.[1031A>G]; [1031A>G] or p.[Glu344Gly]; [Glu344Gly]	*ACADS*: c.[1031A>G]; [1031A>G] or p.[Glu344Gly]; [Glu344Gly]	SCADD
P6	Male	C4: 1.25	C4: 1.59	*ACADS*: [c.431C>T] or p.[Thr144Ile]	*ACADS*: c.[431C>T]; [1156C>T] or p.[T144Ile]; [Arg386Cys]	SCADD
P7	Male	C4: 1.23	C4: 1.15	*ACAD8*: c.[286G>A] or p.[Gly96Ser]; ATP7B: c.[3443T>C]; [3443T>C] or p.[Ile1148Thr]; [Ile1148Thr]	*ACAD8*: c.[286G>A]; [235C>G] or p.[Gly96Ser]; [Arg79Gly]; ATP7B: c.[3443T>C]; [3443T>C] or p.[Ile1148Thr]; [Ile1148Thr]	IBDHD/WD
P8	Female	C4: 2.28	C4: 1.49	*ACAD8*: c.[286G>A] or p.[Gly96Ser]	*ACAD8*: c.[286G>A]; [284T>C] or p.[Gly96Ser]; [Val95Ala]	IBDHD
P9	Male	C4: 1.06	C4: 1.16	*ACAD8*: c.[286G>A] or p.[Gly96Ser]	*ACAD8*: c.[286G>A]; [753_754del] or p.[Gly96Ser]; [Ala252Cysfs*60]	IBDHD
P10	Male	C5: 0.76	C5: 1.05	*IVD*: c.[631A>G]; [631A>G] or p.[Thr211Ala]; p.[Thr211Ala]	*IVD*: c.[631A>G]; [631A>G] or p.[Thr211Ala]; p.[Thr211Ala]	IVA
P11	Male	C5: 0.6	C5: 1.39	*ACADSB*: c.[1165A>G]; [1165A>G] or p.[Met389Val]; [Met389Val]	*ACADSB*: c.[1165A>G]; [1165A>G] or p.[Met389Val]; [Met389Val]	2-MBAD
P12	Female	C5: 0.81	C5: 0.79	*ACADSB*: c.[1165A>G]; [1165A>G] or p.[Met389Val]; [Met389Val]	*ACADSB*: c.[1165A>G]; [1165A>G] or p.[Met389Val]; [Met389Val]	2-MBAD
P13	Male	C5: 0.81	C5: 0.45	*ACADSB*: c.[275C>G]; [655G>A] or p.[Ser92*]; [Val219Met]	*ACADSB*: c.[275C>G]; [655G>A] or p.[Ser92*]; [Val219Met]	2-MBAD
P14	Female	C5DC: 2.87	C5DC: 2.46	*GCDH*: c.[1244-2A>C]; [1244-2A>C]	*GCDH*: c.[1244-2A>C]; [1244-2A>C]	GA-I
P15	Male	C5DC: 2.15	C5DC: 2.24	*GCDH*: c.[1244-2A>C]	*GCDH*: c.[1244-2A>C]; [636-4_639del]	GA-I
P16	Female	C5OH: 6.97	C5OH: 8.71	*MCCC1*: c.[863A>G]; [313C>T] or p.[Glu288Gly]; [Gln105*]	*MCCC1*: c.[863A>G]; [313C>T] or p.[Glu288Gly]; [Gln105*]	3-MCCD
P17	Male	C5OH: 3.65	C5OH: 2.84	Negative	*MCCC1*: c.[980C>G]; [196C>T] or p.[Ser327*]; [Arg66Cys]	3-MCCD
P18	Male	C5OH: 9.53	C5OH: 12.85	Negative	*MCCC2*: c.[351_353del]; [1488G>C] or p.[G118del]; [Gln496His]	3-MCCD

NBS: newborn screening; C3: propionylcarnitine; C4: butyrylcarnitine; C5: isovalerylcarnitine; C5DC: glutarylcarnitine; C5OH: 3-hydroxyisovalerylcarnitine; WD: Wilson’s disease; MMA: methylmalonic acidemia; SCADD: short-chain acyl-CoA dehydrogenase deficiency; IBDHD: isobutyryl-CoA dehydrogenase deficiency; IVA: isovaleric acidemia; 2-MBAD: 2-methylbutyryl-CoA dehydrogenase deficiency; GA-I: glutaric acidemia type 1; 3-MCCD: 3-methylcrotonyl-CoA carboxylase deficiency. Cutoff value of NBS: C3: 0.3–4.5 μmol/L: C4: 0.08–0.45 μmol/L: C5: 0.03–0.35 μmol/L: C5DC: 0.03–0.3 μmol/L: C5OH: 0.0.7–0.65 μmol/L. * The retest concentrations within the cutoff value are given in bold.

Among these patients, three with MMA were compound heterozygous for *MMUT*, two with SACDD were homozygous or compound heterozygous for *ACADS*, two with IBDHD were compound heterozygous for c.286G>A (p.Gly96Ser) in *ACAD8*, One patients (P7) were homozygous for c.3443T>C (p.Ile1148Thr) in *ATP7B*, one with IVA was homozygous for c.631A>G (p.Thr211Ala) in *IVD*, three with 2-MBAD were homozygous or compound heterozygous for *ACADSB*, two with GA-I were homozygous or compound heterozygous for *GCDH*, and three with 3-MCCD were compound heterozygous for *MCCC1* or *MCCC2*. Information on combined genetic screening and confirmatory genetic diagnosis for all 18 patients is summarized in Table 2.

### 3.3. Newborns with False-Positive Screening Results

Thirty-seven newborns with positive biochemical and/or genetic screening results were ultimately confirmed as carriers or unaffected individuals through confirmatory genetic testing (Table 3). Among these, three newborns (No. 18–20) had elevated C5 levels on repeat DBS testing but negative results on combined genetic screening. Follow-up showed that their biomarkers had returned to normal ranges. Additionally, five newborns (No. 21–23, 34, and 35) showed significantly elevated C5OH levels during the recall review, which were attributed to maternal 3-MCCD (Table 3).

## 4. Discussion

Newborn genetic screening holds significant promise, and population-based NBS programs utilizing genomic sequencing as a first-tier screening strategy have already been initiated in several developed countries, such as the BabySeq, Early Check, Screen4Care, and Genomic England programs [28,29,30,31]. However, the implementation of newborn genetic screening in the general neonatal population still faces many challenges, including cost, infrastructure, specialized expertise, data protection, and ethical considerations [32]. Although such screening may enhance detection capabilities, it benefits only a small proportion of patients. Importantly, interpreting identified variants in the absence of biochemical and clinical information remains challenging, especially for variants of uncertain significance. Therefore, the necessity of implementing universal genetic screening for the general neonatal population requires further systematic validation.

Recently, an increasing number of studies have proposed integrating NGS as an additional platform to complement traditional biochemical screening. By combining genetic information with biochemical screening results, the detection capability and diagnostic efficiency of newborn screening can be substantially improved. Chan et al. [33] performed NGS as a second-tier test for six IMDs, which not only reduced the FPR from 0.38% to 0.017% but also successfully identified two false-negative cases of citrin deficiency. Fecarotta et al. [20] proposed that combining biochemical profiling and genetic sequencing analyses could achieve a 100% diagnostic rate, indicating that both biochemical and genetic information are indispensable for accurate classification and diagnosis. However, incorporating NGS as a second-tier genetic test also has potential drawbacks. For instance, a small number of patients with negative biochemical screening results may remain undetected using this integrated screening strategy. Therefore, regardless of the integrated screening strategy implemented in a newborn screening program, its benefits must be carefully weighed against potential risks in terms of screening efficiency, accuracy, and cost-effectiveness.

In this study, we evaluated the use of NGS as a combined genetic screening method for newborns with positive NBS results suggestive of OADs. Although biochemical screening currently has a cost advantage, genetic screening can shorten the diagnostic turnaround time for diagnosis, thereby alleviating parental anxiety and facilitating early diagnosis and timely treatment. As the cost of NGS continues to decline, this combined screening strategy may better support NBS programs. Our results showed that the PPV for OADs increased from 11.26% (17/151) to 30.91% (17/55) with the implementation of a predesigned NGS panel as a combined genetic test. However, several studies have reported diagnostic yields of approximately 50% to 68% for NGS-based genetic testing in neonates suspected of IMDs [25,34,35]. This relatively high performance is largely attributable to the design of the NGS panels, which often include more prevalent disorders, such as hyperphenylalaninemia and glucose-6-phosphate dehydrogenase deficiency. Recently, a large-scale neonatal genetic screening study in China demonstrated that the PPV for amino acid disorders, OADs, and fatty acid oxidation disorders reached 70.83% when genomic sequencing was used as a first-tier screening test [21,36]. Therefore, the PPV for neonatal IMDs varies substantially depending on the disease spectrum, detection methods, and screening strategies employed.

Multiplex PCR-based NGS involves the design of multiple pairs of primers to amplify multiple target gene regions, and its experimental design is relatively simple. This method offers advantages including simple operation, low cost, and short turnaround time, making it suitable for rapid screening of large sample cohorts. In this study, multiplex PCR-based NGS was applied for combined genetic screening, with an overall turnaround time of seven working days. It enables the quick confirmation for high-risk newborns whose biomarkers exceed high cutoff values. In contrast, traditional MS/MS-based screening requires at least 14 working days to generate genetic reports. Genotyping information for more than half of the patients (9/17; 52.9%) was obtained through combined genetic screening. For these patients, the diagnostic turnaround time has been shortened by at least half, enabling more rapid diagnosis. In comparison, hybrid capture-based NGS captures a wide range of genomic fragments by hybridizing biotin-labeled probes to target regions. This approach provides comprehensive coverage of exons and flanking intronic sequences and is suitable for detecting previously unknown genomic variants and for comprehensive genetic diagnosis. However, there are several limitations, such as complex probe design, higher cost, multiple operational steps, and longer turnaround times.

Because the genetic screening assay only detects known disease-causing variants, rare/novel/VUS variants not included in the panel may escape detection, potentially resulting in false-negative results. In this cohort, two patients (P17 and P18) were ultimately diagnosed with 3-MCCD. Although they remained positive on repeat biochemical testing, their genetic screening results were negative, indicating that genetic screening alone may yield false negatives. Among the three patients with MMA, the C3 level of patient P3 was within the normal range at recall evaluation. This case would likely have been missed by traditional NBS alone but was identified through combined genetic screening. Integrating genetic screening with biochemical testing can effectively reduce the false-negative rate associated with biochemical screening and identify additional subgroups of patients that are undetectable by conventional biochemical screening. Biomarkers of other IMDs like long-chain defects may also return to normal at the recall DBS, the false-negative rate would be reduced if genetic testing was performed. These findings support the integration of genetic screening with MS/MS-based biochemical screening to reduce the risk of missed diagnoses and improve overall screening efficiency.

In addition to OADs, we identified two additional cases of WD using newborn genetic screening, demonstrating its potential to broaden the spectrum of detectable diseases. Because specific screening biomarkers for WD are missing in the neonatal period, most patients are diagnosed clinically after symptom onset. Early studies estimated the global incidence of WD to range from 1/30,000 to 1/10,000, with an ATP7B carrier rate of approximately 1/90 [37]. Recently, several newborn genetic screening studies have shown that the incidence of WD is higher in China, with reported incidences ranging from 1/7147 to 1/3844, and carrier rates ranging from 1/51 to 1/31 [13,26,38]. Newborn genetic screening may therefore provide more accurate epidemiological data and help clarify real the true incidence of WD. In this study, two cases of WD were incidentally identified among newborns who screened positive for OADs. This is unlikely to be related to the screening biomarkers (C3 and C4) but may reflect the relatively high incidence of WD in China. Two homozygous pathogenic variants were discovered in *ATP7B* were identified:, c.2333G>T, one of the most common variants in China, and c.3443T>C, which is more prevalent in Southern China [39]. Both patients are currently undergoing regular follow-up in the hepatology department of our hospital. Early detection and timely intervention may help prevent adverse consequences.

Among the 96 cases with positive biochemical screening but negative genetic screening results, biomarker levels returned to normal during follow-up. It was revealed that 63.58% (96/151) of false positives for OADs would be eliminated if genetic screening was applied in clinical practice. The high FPR of traditional NBS not only causes parental anxiety but also increases unnecessary medical expenses due to repeat testing and follow-ups. Consequently, this combined screening approach represents a practical compromise, in which NGS-based targeted sequencing is applied for suspected cases identified by biochemical screening. To some extent, this alleviates the burden of molecular diagnosis, shortens the turnaround time from screening to diagnosis, and reduces parental anxiety, unnecessary recalls, and follow-up medical costs.

However, this study has several limitations. First, rare/novel/VUS variants not included in the genetic screening panel may escape detection; therefore, genetic screening should be integrated with MS/MS-based screening to minimize missed cases. Second, genetic screening was performed only in high-risk newborns who tested positive on MS/MS screening. Ninety-six of the 151 newborns were excluded because of negative results on repeat biochemical screening and genetic screening. Given the extremely low likelihood of OADs in screen-negative individuals, no further genetic analyses were conducted for these 96 newborns. Third, newborns with slightly elevated NBS results were not selected for combined genetic screening, and their biochemical results normalized during recall. We will install the molecular testing as a second-tier test in the future to decrease false positive, speed up diagnosis, or increase sensitivity. While the cut-off values should be carefully optimized to further reduce the risk of missed cases and improve overall efficiency of NBS. Finally, the sample size was relatively small, and the cutoff values established for genetic screening may not be optimal. Larger-scale studies evaluating the clinical performance and cost-effectiveness of this NGS-based genetic screening are needed.

## 5. Conclusions

In summary, this study demonstrated the application of NGS as a combined genetic screening tool for OADs. Seventeen patients were finally diagnosed with OADs, with a PPV of 30.91%. Incorporating NGS into MS/MS-based newborn screening can effectively enhance screening efficiency by reducing false-positive results and shortening the diagnostic turnaround time. Additionally, two cases of WD were identified using NGS-based genetic screening, highlighting the potential of genetic screening to broaden the spectrum of detectable diseases. These results provide a reference for the application and optimization of newborn genetic screening.

## Figures and Tables

**Table 1:** NBS biomarkers and cutoff values for screen organic acidemias.

NBS Biomarker	Cutoff Value of NBS (μmol/L)	Cutoff Value of Genetic Screening (μmol/L)	Target Disorder
C3	0.3–4.5	>6.5	MMA/PA
C4	0.08–0.45	>0.9	IBDHD
C5	0.03–0.35	>0.6	IVA/2-MBAD
C5DC	0.03–0.3	>0.5	GA-I
C5OH	0.07–0.65	>1.0	3-MCCD

C3: propionylcarnitine; C4: butyrylcarnitine; C5: isovalerylcarnitine; C5DC: glutarylcarnitine; C5OH: 3-hydroxyisovalerylcarnitine; WD: Wilson’s disease; MMA: methylmalonic acidemia; SCADD: short-chain acyl-CoA dehydrogenase deficiency; IBDHD: isobutyryl-CoA dehydrogenase deficiency; IVA: isovaleric acidemia; 2-MBAD: 2-methylbutyryl-CoA dehydrogenase deficiency; GA-I: glutaric acidemia type 1; 3-MCCD: 3-methylcrotonyl-CoA carboxylase deficiency.

**Table 3:** Newborns with screen-positive results using tandem mass spectrometry combined with genetic screening.

No.	Gender	NBS (μmol/L)	Retest (μmol/L) *	Combined Genetic Screening	Confirmatory Genetic Test (Targeted NGS and Sanger Sequencing)
1	Female	C3: 7.80	C3: 0.94	*MMUT*: c.[2080C>T] or p.[Arg694Trp]	*MMUT*: c.[2080C>T] or p.[Arg694 Trp]
2	Female	C3: 8.21	C3: 0.66	*ACADSB*: c.[655G>A] or p.[Val219Met]	*ACADSB*: c.[655G>A] or p.[Val219Met]
3	Female	C3: 8.13	C3: 0.82	*MMUT*: c.[323G>A] or p.[Arg108His]	*MMUT*: c.[323G>A] or p.[Arg108His]
4	Male	C3: 6.50	C3: 2.87	*ETFDH*: c.[250G>A] or p.[Ala84Thr]	*ETFDH*: c.[250G>A] or p.[Ala84Thr]
5	Male	C3: 7.44	C3: 0.51	*GCDH*: c.[1244-2A>C]	*GCDH*: c.[1244-2A>C]
6	Male	C4: 0.96	C4: 0.32	*ACADS*: c.[1031A>G] or p.[Glu344Gly]	*ACADS*: c.[1031A>G] or p.[Glu344Gly]
7	Male	C4: 0.91	**C4: 0.79**	*ACADS*: c.[1138C>T] or p.[Arg380Trp]	*ACADS*: c.[1138C>T] or p.[Arg380Trp]
8	Male	C4: 1.27	**C4: 0.46**	*ACADS*: c.[815G>A] or p.[Arg272His]	*ACADS*: c.[815G>A] or p.[Arg272His]
9	Male	C4: 0.98	**C4: 0.56**	*ACADS*: c.[1195C>T] or p.[Arg399Trp]	*ACADS*: c.[1195C>T] or p.[Arg399Trp]
10	Female	C4: 0.96	**C4: 0.52**	*ACADS*: c.[1031A>G] or p.[Glu344Gly]	*ACADS*: c.[1031A>G] or p.[Glu344Gly]
11	Female	C4: 0.94	**C4: 0.51**	*ACADSB*: c.[1165A>G] or p.[Met389Val]	*ACADSB*: c.[1165A>G] or p.[Met389Val]
12	Female	C5: 0.63	**C5: 0.56**	*ACADSB*: c.[275C>G] or p.[Ser92*]	*ACADSB*: c.[275C>G] or p.[Ser92*], *IVD*: c.[457-3_457-2delinsGG]
13	Female	C5: 0.74	C5: 0.28	*HMGCS2*: c.[520T>C] or p.[Phe174Leu]	*HMGCS2*: c.[520T>C] or p.[Phe174Leu], *G6PD*: c.[1376G>T] or p.[Arg459Leu]
14	Female	C5: 0.69	C5: 0.18	*GCDH*: c.[1244-2A>C]	*GCDH*: c.[1244-2A>C]
15	Female	C5: 0.65	C5: 0.21	*HMGCS2*: c.[520T>C] or p.[Phe174Leu]	*HMGCS2*: c.[520T>C] or p.[Phe174Leu]
16	Male	C5: 0.70	C5: 0.35	*ACADSB*: c.[1165A>G] or p.[Met389Val]	*ACADSB*: c.[1165A>G] or p.[Met389Val]
17	Male	C5: 0.69	C5: 0.27	*IVD*: c.[631A>G] or p.[Thr211Ala]	*IVD*: c.[631A>G] or p.[Thr211Ala]
18	Male	C5: 1.22	**C5: 1.10**	Negative	Negative
19	Male	C5: 3.76	**C5: 0.71**	Negative	Negative
20	Male	C5: 1.79	**C5: 0.51**	Negative	*ETFDH:* c.[1252del] or p.[Leu418*]
21	Female	C5OH: 4.63	**C5OH: 3.77**	*MCCC1*: c.[863A>G] or p.[Glu288Gly]	*MCCC1*: c.[863A>G] or p.[Glu288Gly]
22	Male	C5OH: 9.57	**C5OH: 7.53**	*MCCC1*: c.[863A>G] or p.[Glu288Gly] *HMGCS2*: c.[520T>C] or p.[Phe174Leu]	*MCCC1*: c.[863A>G] or p.[Glu288Gly] *HMGCS2*: c.[520T>C] or p.[Phe174Leu]
23	Male	C5OH: 9.97	**C5OH: 33.42**	*MCCC1*: c.[1331G>A] or p.[Arg444His]	*MCCC1*: c.[1331G>A] or p.[Arg444His]
24	Female	C5OH: 1.03	**C5OH: 1.13**	*MCCC1*: c.[1331G>A] or p.[Arg444His]	*MCCC1*: c.[1331G>A] or p.[Arg444His]
25	Male	C5OH: 1.50	**C5OH: 1.05**	*MCCC1*: c.[1331G>A] or p.[Arg444His]	*MCCC1*: c.[1331G>A] or p.[Arg444His]
26	Male	C5OH: 1.16	**C5OH: 0.70**	*MCCC1*: c.[863A>G] or p.[Glu288Gly]	*MCCC1*: c.[863A>G] or p.[Glu288Gly]
27	Male	C5OH: 1.07	**C5OH: 0.80**	*MCCC1*: c.[863A>G] or p.[Glu288Gly]	*MCCC1*: c.[863A>G] or p.[Glu288Gly]
28	Male	C5OH: 1.28	**C5OH: 0.80**	*MCCC1*: c.[863A>G] or p.[Glu288Gly]	*MCCC1*: c.[863A>G] or p.[Glu288Gly] *ETFDH*: c.[250G>A] or p.[Ala84Thr]
29	Female	C5OH: 1.13	**C5OH: 0.76**	*MCCC1*: c.[863A>G] or p.[Glu288Gly]	*MCCC1*: c.[863A>G] or p.[Glu288Gly]
30	Male	C5OH: 1.27	**C5OH: 0.80**	*MCCC1*: c.[863A>G] or p.[Glu288Gly]	*MCCC1*: c.[863A>G] or p.[Glu288Gly]
31	Male	C5OH: 1.05	**C5OH: 0.84**	*MCCC1*: c.[639+2T>A] or p.[Ser164Argfs*3]	*MCCC1*: c.[639+2T>A] or p.[Ser164Argfs*3]
32	Male	C5OH: 1.41	**C5OH: 0.90**	*MCCC1*: c.[1331G>A] or p.[Arg444His]	*MCCC1*: c.[1331G>A] or p.[Arg444His]
33	Male	C5OH: 1.11	**C5OH: 0.72**	*ETFDH*: c.[770A>G] or p.[Tyr257Cys]	*ETFDH*: c.[770A>G] or p.[Tyr257Cys]
34	Female	C5OH: 17.48	**C5OH: 8.25**	Negative	*MCCC2*: c.[351_353del] or p.[Gly118del]
35	Male	C5OH: 4.60	**C5OH: 3.40**	Negative	*MCCC2*: c.[351_353del] or p.[Gly118del]
36	Female	C5OH: 1.10	**C5OH: 0.99**	Negative	*MCCC1*: c.[1630del] or p.[Arg544Aspfs*2]
37	Male	C5OH: 1.00	**C5OH: 0.74**	Negative	Negative

NBS, newborn screening; C3, propionylcarnitine; C4, butyrylcarnitine; C5, isovalerylcarnitine; C5DC, glutarylcarnitine; C5OH, 3-hydroxyisovalerylcarnitine. Cutoff value of NBS: C3: 0.3–4.5 μmol/L, C4: 0.08–0.45 μmol/L, C5: 0.03–0.35 μmol/L, C5DC: 0.03–0.3 μmol/L, C5OH: 0.07–0.65 μmol/L. * The retest concentrations exceed the cutoff value are given in bold.

## Data Availability

Data included in article/Supplementary Material/reference in article. The datasets used and analyzed in this study are available from the corresponding author upon reasonable request.

## Supplementary Materials

The following supporting information can be downloaded at: https://www.mdpi.com/article/10.3390/ijns12010018/s1, Table S1: The list of sreened diseases, genes, and variations; Table S2: Targeted diseases and genes for confirmatory genetic analysis.

## Abbreviations

NBS: newborn screening; MS/MS: tandem mass spectrometry; DBS: dried blood spot; IMDs: inherited metabolic disorders; OADs: organic acidemias; FPR: false-positive rate; PPV: positive predictive value; NGS: next-generation sequencing; C3: propionylcarnitine; C4: butyrylcarnitine; C5: isovalerylcarnitine; C5DC: glutarylcarnitine; C5OH: 3-hydroxyisovalerylcarnitine; WD: Wilson’s disease; MMA: methylmalonic acidemia; SCADD: short-chain acyl-CoA dehydrogenase deficiency; IBDHD: isobutyryl-CoA dehydrogenase deficiency; IVA: isovaleric acidemia; 2-MBAD: 2-methylbutyryl-CoA dehydrogenase deficiency; GA-I: glutaric acidemia type 1; 3-MCCD: 3-methylcrotonyl-CoA carboxylase deficiency.
